# Supplementary material for: Nucleus reuniens transiently synchronizes memory networks at beta frequencies
Source: Nat Commun. 2023 Jul 19;14:4326. doi: 10.1038/s41467-023-40044-z (PMC10356781; doi:10.1038/s41467-023-40044-z)
Supplement: Supplementary file 1 — Supplementary Information [file 41467_2023_40044_MOESM1_ESM.pdf]

SUPPLEMENTARY INFORMATION

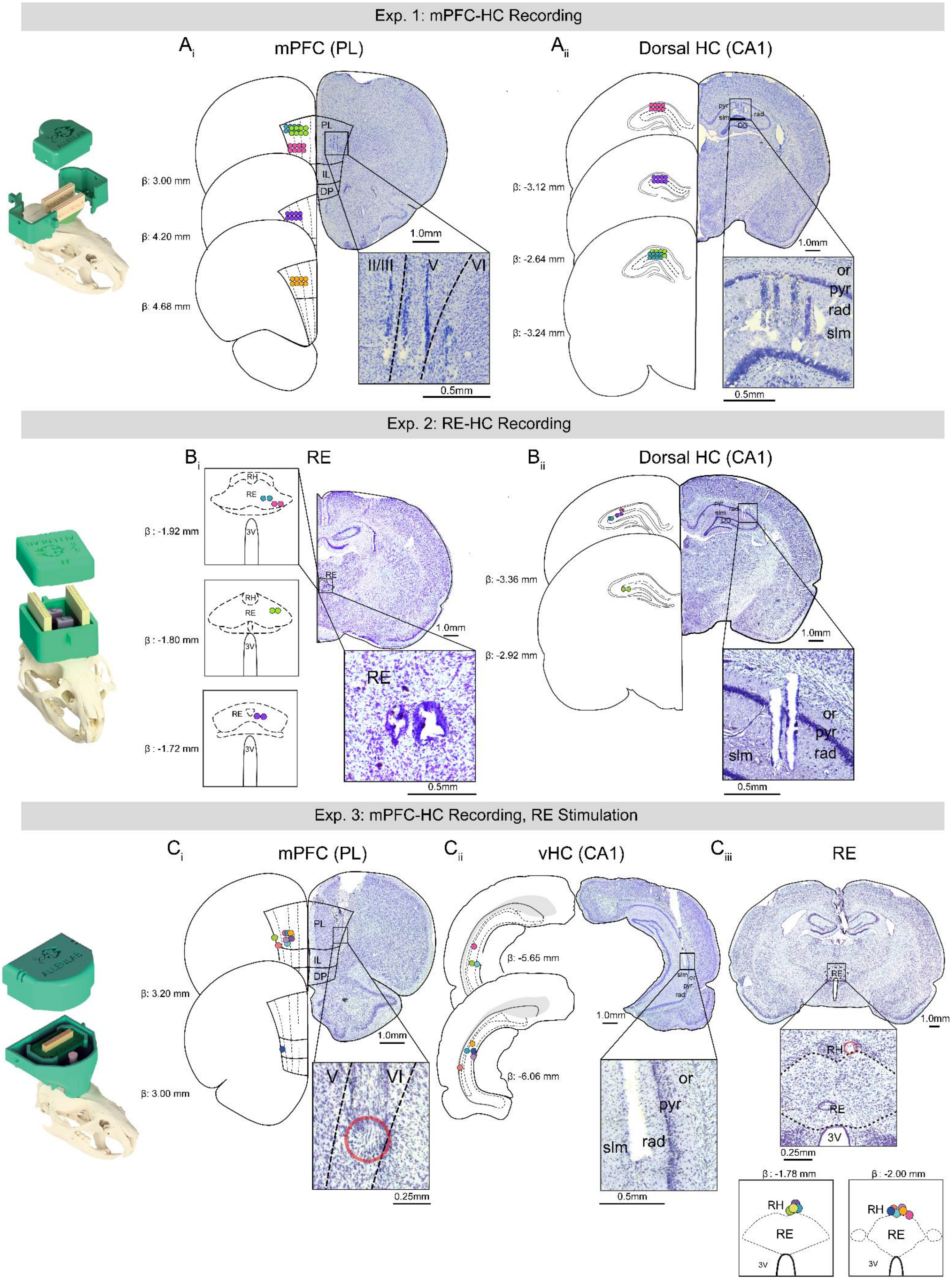

Supplementary Figure Fig. 1: Electrode placements for all rats, Related to Figures 1, 2, and 3. A) Representative Nissl-stained images are shown for rats used in Experiment 1 with probes targeting **A_i)** mPFC (n = 5) and **A_{ii})** dHC(n = 4). Probes were implanted on the right side. Marking lesions were located across layers in the prelimibic cortex and slm/rad. On the left, a schematic representation of the targeted region and probe placement using Paxinos & Watson[66] brain overlays with permission is shown for all rats. The skull image was modified and reproduced from Jayachandran and Allen[76]. Histological procedures were repeated for each rat and represented with different colored circles to indicate their exact location. **B)** Representative Nissl-stained image is shown for rats used in Experiment 2 with probes targeting **B_i)** RE (n = 4) and **B_{ii})** dHC(n = 4). Probes were implanted on the right side and the zoomed-in image shows that the marking lesions were located toward the lateral aspects of RE and slm/rad in CA1. On the left, a schematic representation of the targeted region and probe placement using Paxinos & Watson brain overlays with permission[66] is shown for all rats. Histological procedures were repeated for each rat and represented with different colored circles to indicate their exact location **C)** Representative Nissl-stained image is shown for rats used in Experiment 3 with ss targeting **C_i)** mPFC and **C_{ii})** vHC and **C_{iii})** optrode placement in RE (n = 5 experimental; n = 5 control). A schematic representation of the targeted region and probe placement using Paxinos & Watson[66] brain overlays with permission is shown for all rats. Histological procedures were repeated for each rat and represented with different colored circles to indicate their exact location. Abbreviations: mPFC, medial prefrontal cortex; HC, hippocampus; dHC, dorsal hippocampus; vHC, ventral hippocampus; RE, nucleus reuniens; RH, rhomboid nucleus; slm, stratum lacunosum-moleculare; rad, radiatum; pyr, pyramidal; or, stratum oriens; CA1, Cornus Ammonis 1; PL, prelimbic; IL, infralimbic; DP, dorasl peduncular cortex; ss, saggital sinus. Source data are provided as a Source Data file.

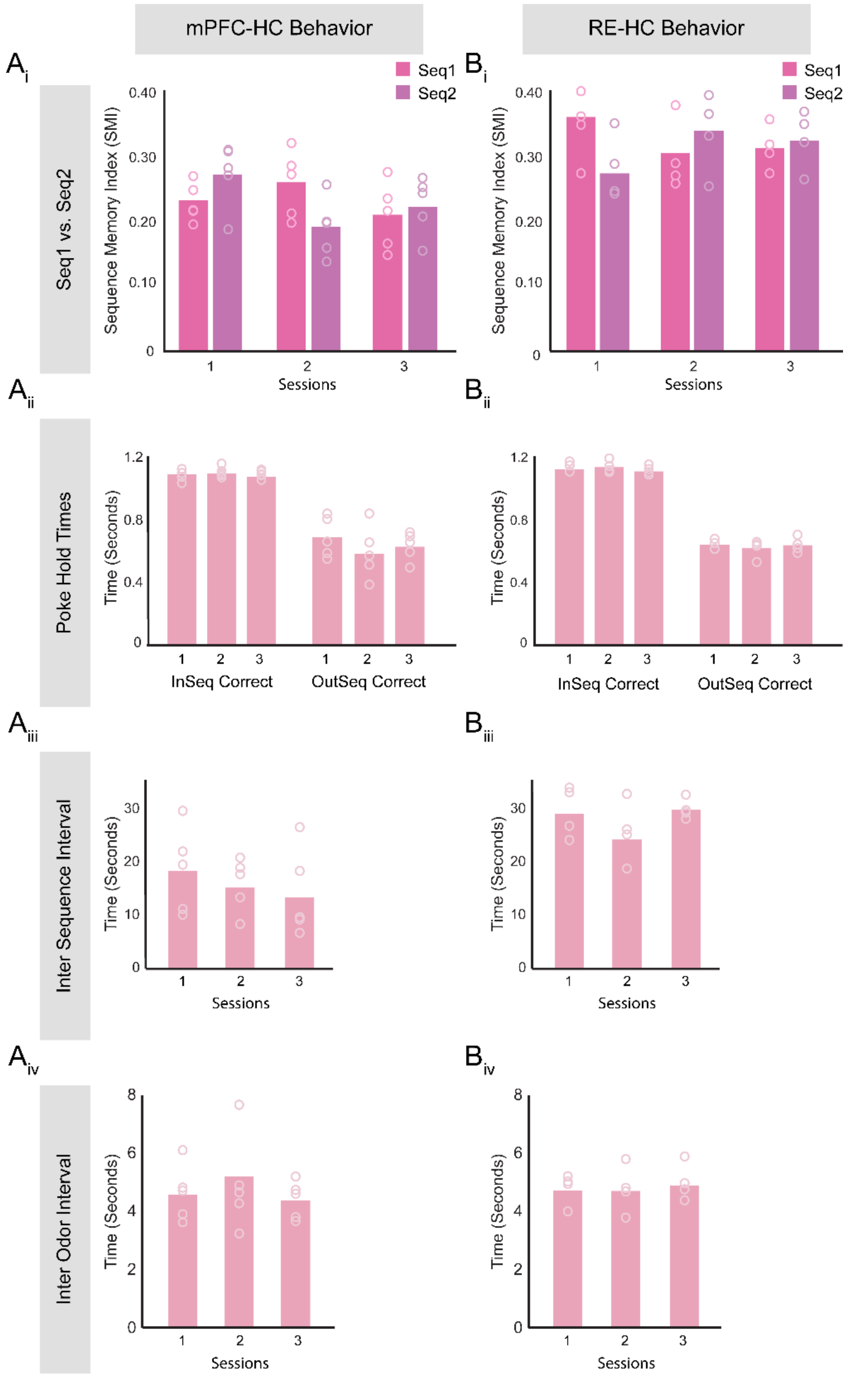

Supplementary Figure Fig. 2: Sequence memory and non-mnemonic aspects of behavior did not differ across sessions, Related to Figure 1 and 2. A) Rat behavior on the sequence memory task used in the mPFC-HC experiment. We used a one-way ANOVA with Bonferroni correct for the following analysis. **A_i)** The SMI was not significantly different across sessions ($F_{(2,12)} = 2.40$, $p = 0.13$). Moreover, no significant differences were observed between sequence 1 and sequence 2 across 3 sessions (Session 1: $t_{(4)} = -1.62$, $p = 0.14$; Session 2: $t_{(4)} = 1.85$, $p = 0.10$; Session 3: $t_{(4)} = -0.30$, $p = 0.77$). **A_{ii})** No significant difference was detected across the 3 sessions for $InSeq_{correct}$ poke times ($F_{(2,12)} = 0.34$, $p = 0.72$) and $OutSeq_{correct}$ poke times($F_{(2,12)} = 0.42$, $p = 0.67$), indicating that nose-poke behavior was not affected by sessions. **A_{iii})** ISI did not differ significantly across sessions ($F_{(2,12)} = 0.96$, $p = 0.41$), suggesting that rats in all 3 conditions ran at similar rates between sequences. **A_{iv})** IOI did not significantly differ across sessions ($F_{(2,12)} = 0.62$, $p = 0.55$), suggesting that rats collected water rewards and engaged odors at similar rates. **B)** Rat behavior on the sequence memory task used in the RE-HC experiment. We used a one-way ANOVA with Bonferroni correct for the following analysis. **B_i)** The SMI was not significantly different across sessions ($F_{(2,11)} = 0.04$, $p = 0.96$). No significant differences were detected between sequence 1 and sequence 2 over the 3 sessions (Session 1: $t_{(3)} = 1.75$, $p = 0.18$; Session 2: $t_{(3)} = -1.09$, $p = 0.36$; Session 3: $t_{(3)} = -0.51$, $p = 0.65$). **B_{ii})** No significant difference was detected across sessions for $InSeq_{correct}$ poke times ($F_{(2,11)} = 0.79$, $p = 0.49$) and $OutSeq_{correct}$ poke times ($F_{(2,11)} = 0.44$, $p = 0.66$), indicating that nose-poke behavior was not affected by session. **B_{iii})** ISI did not differ significantly between sessions ($F_{(2,11)} = 1.41$, $p = 0.29$). **B_{iv})** IOI did not differ significantly across sessions ($F_{(2,11)} = 0.12$, $p = 0.89$). All data are represented as mean ± SEM; * $p < 0.05$; ns, not significant. Abbreviations: mPFC, medial prefrontal cortex; HC, hippocampus; $InSeq_{correct}$, in-sequence correct; $OutSeq_{correct}$, out-of-sequence correct; ISI, inter sequence interval; IOI, inter odor interval; SMI, sequence memory index. Source data are provided as a Source Data file.

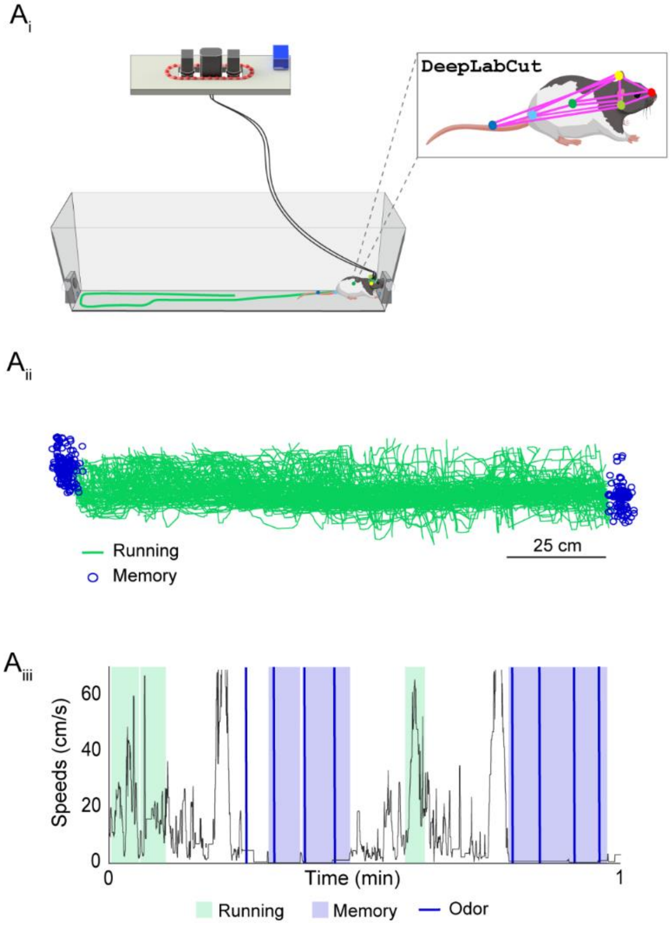

Supplementary Figure Fig. 3: Behavioral conditions obtained using DeepLabCut for sequence memory task for separation in electrophysiological analyses, Related to Figure 1, 2, and 4. Behavioral tracking was achieved using a deep machine learning algorithm from training datasets to achieve quantifiable and accurate animal pose estimation (www.mackenziemathislab.org/deeplabcut). This approximation was further filtered using custom-written scripts in MATLAB 2021a. **A_i)** Rat in sequence memory task with behavioral tracking markers for trained and learned body parts (nose, left and right ears, center of gravity, tail start, and middle of tail; inset). The rat image was modified and reproduced from Jayachandran and Allen[76].**A_{ii})** Representative processed trajectories from 1 rat showing active running in between odors (represented in green), and odor sampling on correct trials (memory) occurring during stationary positions (represented in blue). **A_{iii})** Sample activity plot (~1 min) with filtered speeds during the sequence memory task. Running and stationary bouts are highlighted in green and blue, respectively, as well as odor trial times (blue lines).

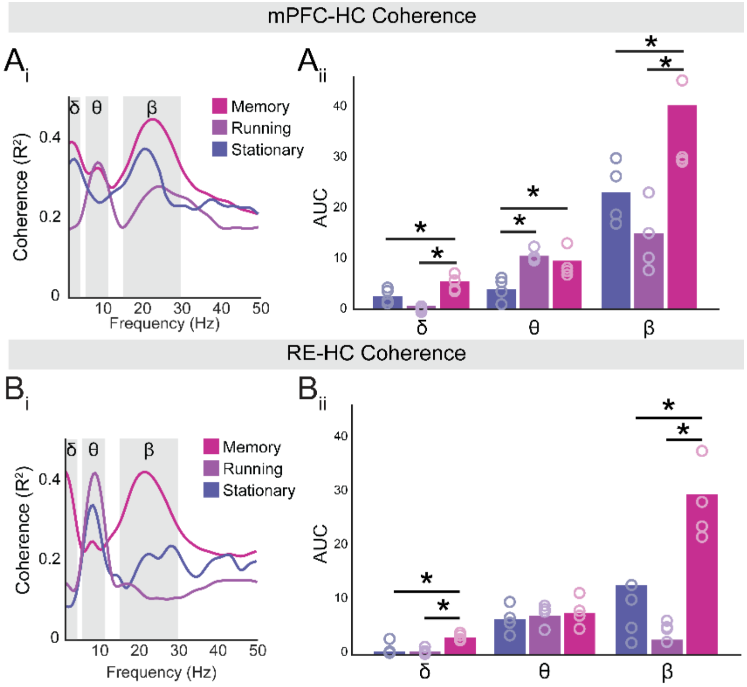

Supplementary Figure Fig. 4: Delta, theta, and beta during different behavioral conditions, Related to Figure 1 and 2. Ai) mPFC-HC coherence showing memory, running, and stationary periods. During memory, both delta and beta increase while theta remains the same. During stationary periods (separate from stationary during the sequence trials) theta decreases while delta and beta increase in coherence, and all 3 frequency bands had lower coherence compared to memory periods. Finally, during running, theta coherence increased while delta and beta decreased. **Aii)** We used a one-way ANOVA with Bonferroni correct for the following analysis. Delta was significantly different between the 3 behavioral conditions ($F_{(2,11)}$ = 12.46, p = 3.0x10^{-3}). Theta was significantly different between the 3 behavioral conditions ($F_{(2,11)}$ = 6.96, p = 0.02). Beta was significantly different between the 3 behavioral conditions ($F_{(2,11)}$ 8.38, p = 0.01). **Bi)** RE-HC coherence showing memory, running, and stationary periods. During memory, both delta and beta increased while theta decreased. During stationary periods (separate from stationary during the sequence trials), theta increased while beta showed a moderate increase in coherence. Finally, during running, theta coherence increased while delta and beta decreased. **Bii)** We used a one-way ANOVA with Bonferroni correct for the following analysis. Delta was significantly different between the 3 behavioral conditions ($F_{(2,11)}$ =11.52, p = 3.0x10^{-3}). Theta was not significantly different between the 3 behavioral conditions ($F_{(2,11)}$ = 0.87, p = 0.45). Beta was significantly different between the 3 behavioral conditions ($F_{(2,11)}$ 43.68, p = 2.3x10^{-5}). All data are represented as mean ± SEM; * p < 0.05; ns, not significant. Abbreviations: mPFC, medial prefrontal cortex; HC, hippocampus; RE, nucleus reuniens; δ, delta; θ, theta; β, beta. Source data are provided as a Source Data file.

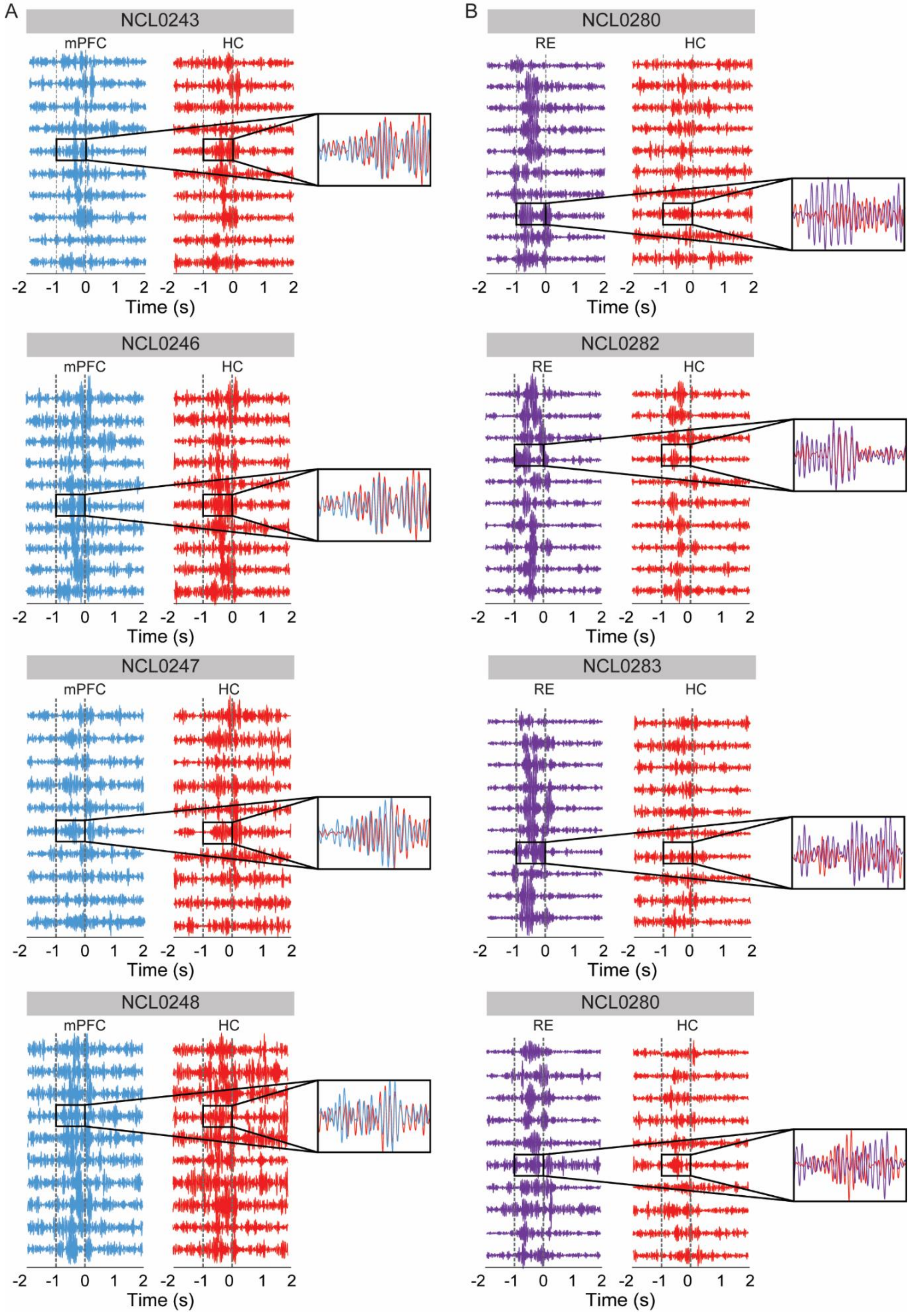

Supplementary Figure Fig. 5. Sample beta-filtered voltage traces during sequence trials, Related to Figure 1 and 2. Bandpass-filtered beta during sequence trials for a subsample of InSeq trials across rats in both groups (mPFC-HC and RE-HC). **A)** Prefrontal cortex and hippocampus showed clear beta synchorny across trials in all rats. **B)** RE beta bursts occured earlier and had a larger amplitude compared with the hippocampus. Abbreviations: InSeq, in-sequence; mPFC, medial prefrontal fortex; HC, hippocampus; RE, nucleus reuniens; NCLXXXX, rat ID.

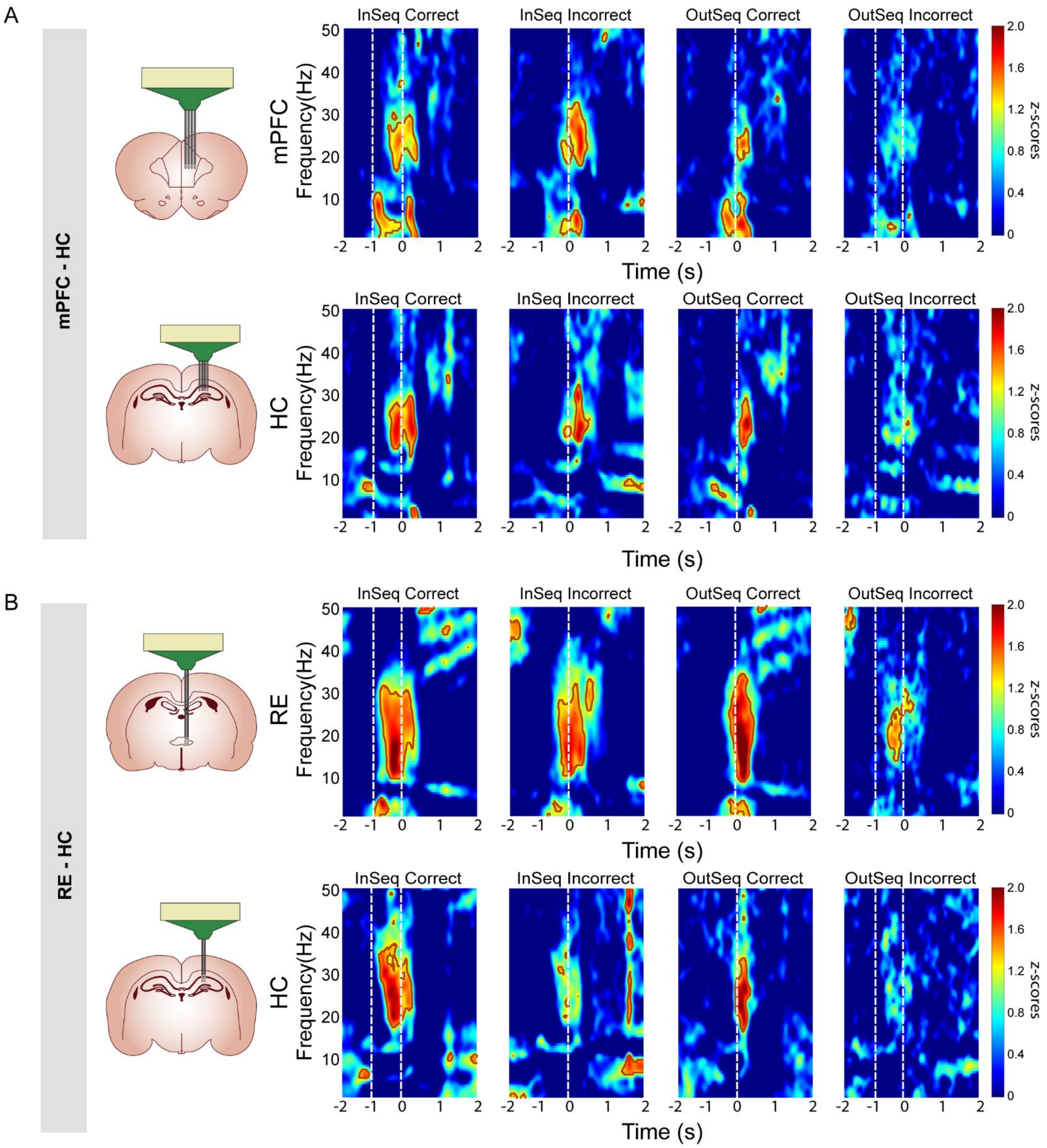

Supplementary Figure Fig. 6: Prefrontal cortex, RE, and hippocampus perievent spectrograms during the sequence memory task, Related to Figure 1 and 2. A) mPFC and HC perievent spectrograms across different trial types aligned to poke out. Beta can be seen in both mPFC and HC. The significant areas (one sample z-tests) where $p < 0.05$ are outlined (brick color). OutSeq trials did not show significant beta in mPFC or HC rats. **B)** RE and HC perievent spectrograms across different trial types aligned to poke out. Beta can be seen in both RE and HC. The significant areas (one sample z-tests) are outlined where $p < 0.05$ (brick color). HC OutSeq incorrect trials failed to show significant beta. Brain schematics in A & B are originals created using Paxinos and Watson outlines with permision[66]. Abbreviations: mPFC, medial prefrontal cortex; HC, hippocampus; RE, nucleus reuniens; InSeq, in sequence; OutSeq, out of sequence; $InSeq_{correct}$, in-sequence correct, $InSeq_{incorrect}$, in-sequence incorrect, $OutSeq_{correct}$, out-of-sequence correct, $OutSeq_{incorrect}$, out-of-sequence incorrect.

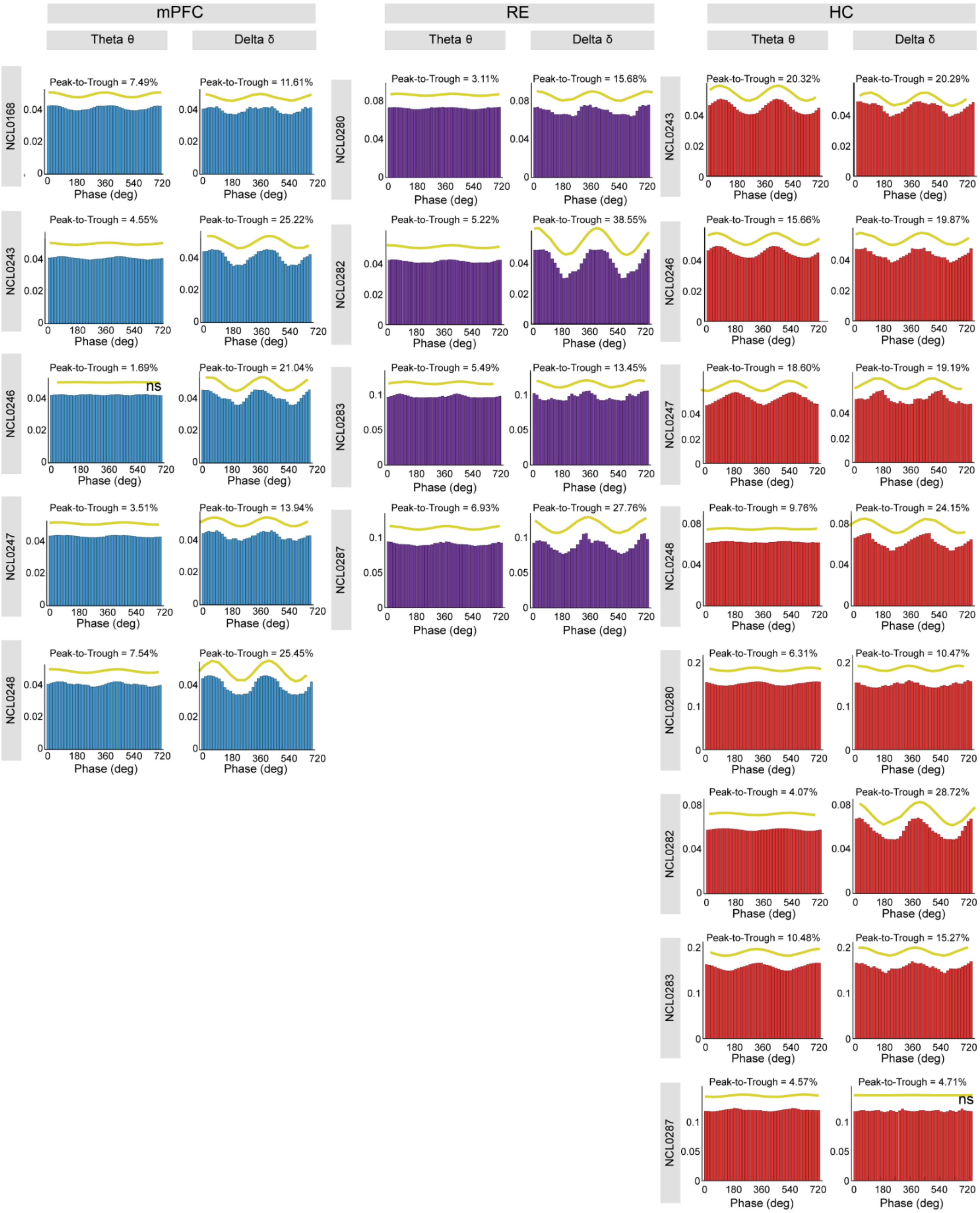

Supplementary Figure Fig. 7. Average beta amplitude-phase coupling with theta and delta during sequence memory trials, Related to Figure 3. Phase-amplitude plots showing the mean beta amplitudes along the phase of theta or delta rhythms recorded on the same electrodes. Subjects were averaged across sessions within regions of interest (mPFC, blue; RE, purple; HC, red). Bar graphs show magnitude of beta amplitude at a phase degree with a fitted sine wave (yellow) and calculated percentage peak-to-trough distance on top. In all but 2 subjects (indicated with ns), a significant relationship between fitted sine wave and plotted data with $p < 0.01$ was observed (sine wave fit was tested via a quadratic regression on the F ratio). Abbreviations: ns, not significant. Abbreviations: mPFC, medial prefrontal cortex; HC, hippocampus; RE, nucleus reuniens; ns, not significant; NCLXXXX, rat ID. Source data are provided as a Source Data file.

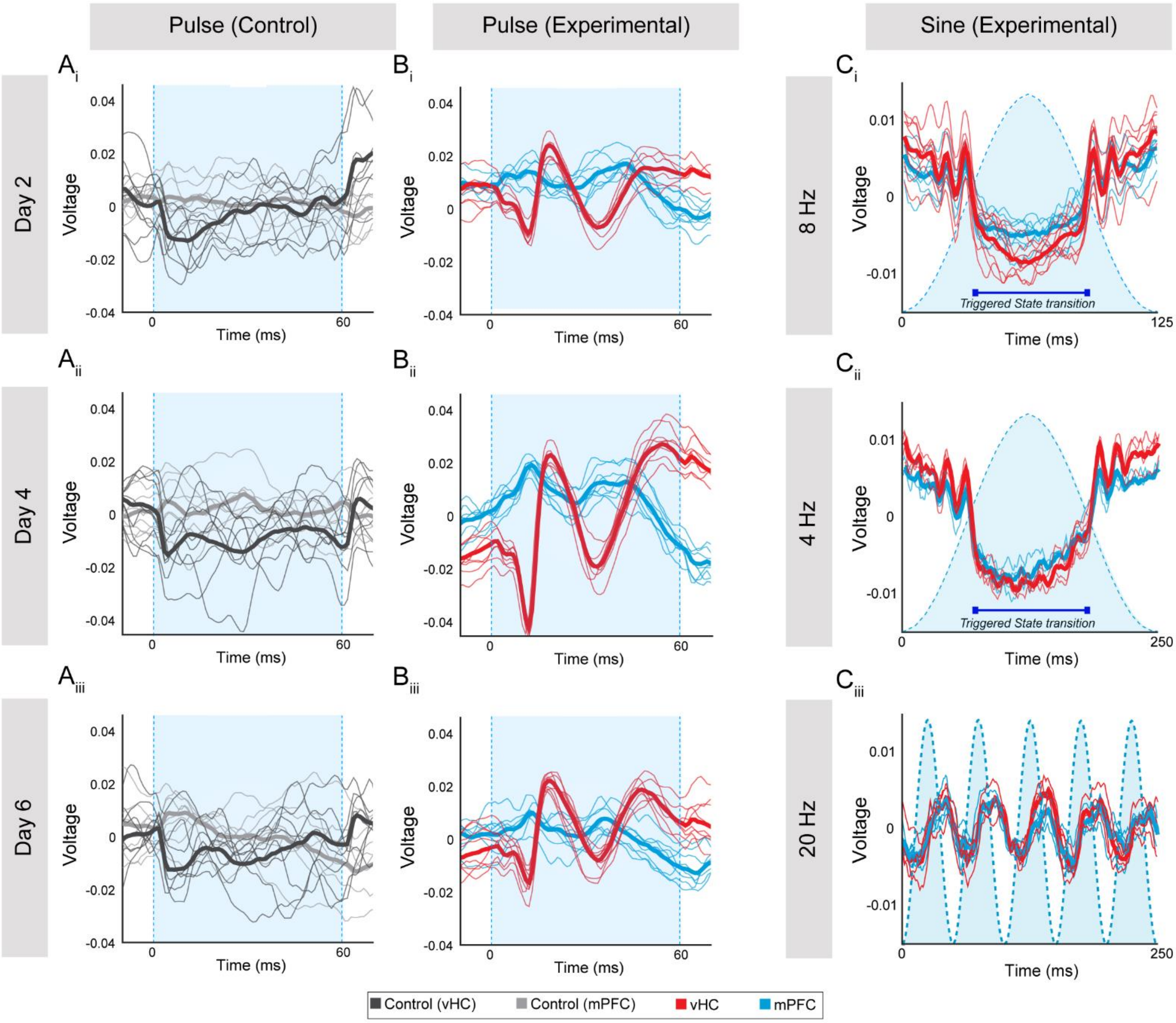

Supplementary Figure Fig. 8: Sample reuniens blue light evoked responses from ventral hippocampus, Related to Figure 4. Stacked mPFC (blue) and vCA1 (red) raw signals from an averaged 1-s period 8-Hz blue light pulse stimulation (60ms each) during the first session (day 2), second session (day 4), and third session (day 6) in viral controls **A_i-A_{iii})**, and experimental (ChR2) subjects **B_i-B_{iii})**. Thick lines represent the mean raw signal across all pulses and thinner lines represent raw signal during each pulse. Note that in the controls the activity remains relatively the same across pulses and days. In contrast, in the ChR2, very strong evoked vCA1 monosynaptic responses (red) are observed consistently between pulses and across days. A similar but less strong response can be observed in mPFC (light blue). **C)** Sine wave stimulations also changed LFP voltage activity in the mPFC (blue) and vCA1 (red) in ChR2 rats. Low (**$C_{i\text{-}ii}$**) and high (**C_{iii}**) sine waves triggered rapid down-state transitions at the rise of the sine wave stimulus, and up-state transitions with the fall of the sine that created rhythmic and phased locked voltage activity in both brain regions. Of note, sinewave stimulations tend to make linear shifts to steadier negative or positive voltage levels in contrast to pulses (Bi-iii) which elicit only rapid potential deflections around ~0 volts but are sufficient to induce changes in the spectral modes within the target regions. Based on this, it is likely that up/down ramping stimuli (saw tooth) can induce rapid state transitions (as reflected in the rising and falling of the light intensity during sinewave stimulations). Overall, this demonstrated that optogenetic excitation of RE neurons effectively elicited electrophysiological responses in the mPFC and hippocampus. Shaded light blue area indicates the duration of the blue light pulse. Abbreviations: mPFC, medial prefrontal cortex; vHC, ventral hippocampus; RE, nucleus reuniens; LFP, local field potential; vCA1, ventral cornus ammonis 1; ChR2, channelrhodopsin. Source data are provided as a Source Data file.

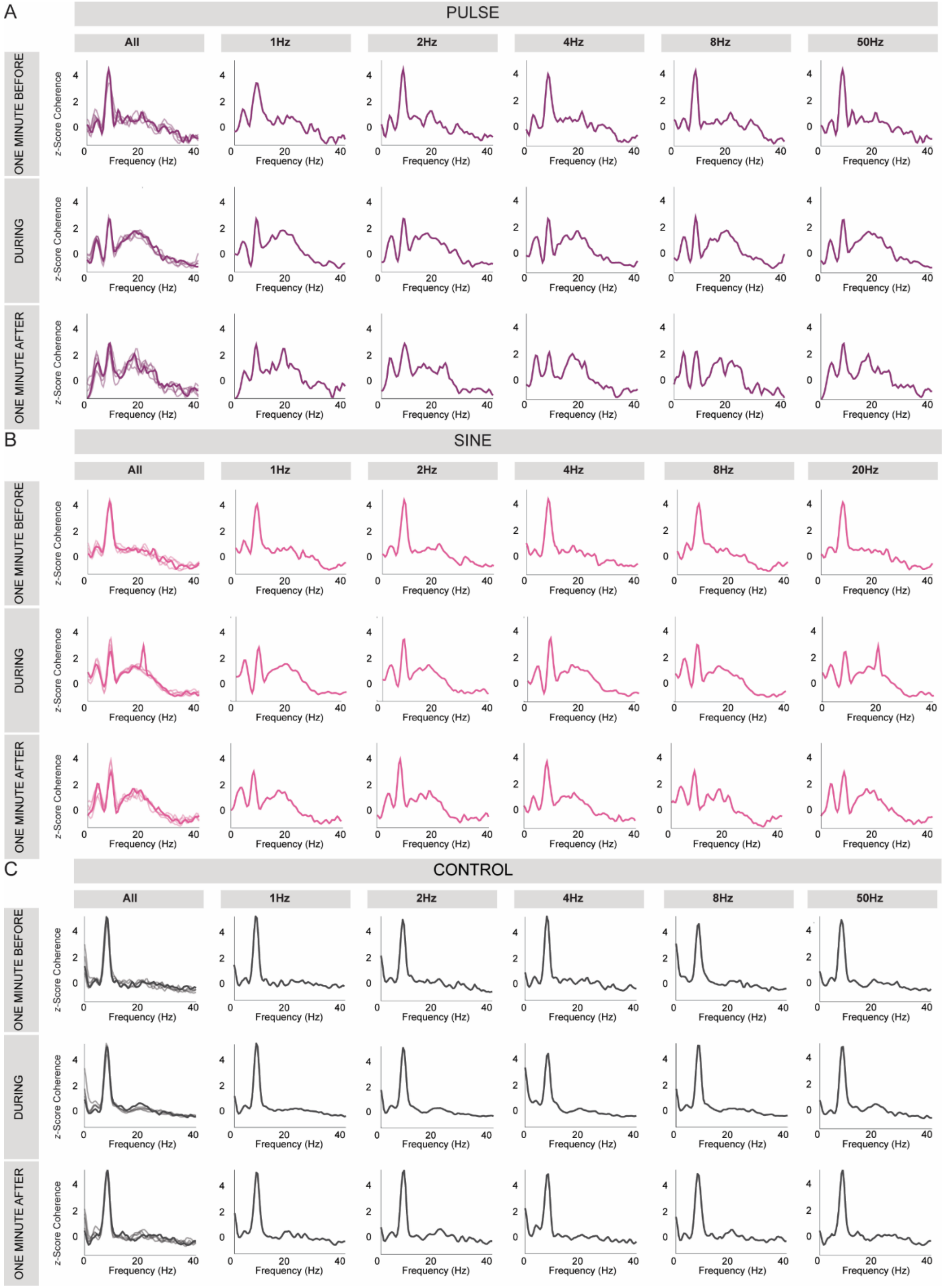

Supplementary Figure Fig. 9. Prefrontal-hippocampal coherence curves were similar during and after RE activation regardless of the frequency of the applied stimulation, Related to Figure 4. mPFC-HC coherence was assessed 1 min before, during, and 1 min after RE optogenetic stimulations. Pulse **(A)** and sine **(B)** stimulation patterns had nearly identical coherence curves (when stacked on top of each other—see All columns) during and after stimulations regardless of the frequency (1, 2, 4, 8, 50 or 20 Hz), with prominent increases in the delta and beta bands and decreases in the theta band. **C)** In control rats (across all time-points), the coherence curves were dominated by theta and totally unaffected by pulse and sine stimulation patterns, as expected. Coherence curves shown are 1 min before stimulation, with all showing a predominant theta coherence. Abbreviations: mPFC, medial prefrontal cortex; HC, hippocampus; RE, nucleus reuniens; ChR2, channelrhodopsin. Source data are provided as a Source Data file.
