# Supplementary material for: Nucleus reuniens transiently synchronizes memory networks at beta frequencies
Source: Nat Commun. 2023 Jul 19;14:4326. doi: 10.1038/s41467-023-40044-z (PMC10356781; doi:10.1038/s41467-023-40044-z)
Supplement: Supplementary file 3 — Reporting Summary [file 41467_2023_40044_MOESM3_ESM.pdf]

nature portfolio

Corresponding author(s): Timothy A. Allen, PhD

Last updated by author(s): Mar 13, 2023

Reporting Summary

Nature Portfolio wishes to improve the reproducibility of the work that we publish. This form provides structure for consistency and transparency in reporting. For further information on Nature Portfolio policies, see our Editorial Policies and the Editorial Policy Checklist.

Statistics

For all statistical analyses, confirm that the following items are present in the figure legend, table legend, main text, or Methods section.

n/a	Confirmed	
☐	☒	The exact sample size (n) for each experimental group/condition, given as a discrete number and unit of measurement
☐	☒	A statement on whether measurements were taken from distinct samples or whether the same sample was measured repeatedly
☐	☒	The statistical test(s) used AND whether they are one- or two-sided<br>*Only common tests should be described solely by name; describe more complex techniques in the Methods section.*
☐	☒	A description of all covariates tested
☐	☒	A description of any assumptions or corrections, such as tests of normality and adjustment for multiple comparisons
☐	☒	A full description of the statistical parameters including central tendency (e.g. means) or other basic estimates (e.g. regression coefficient) AND variation (e.g. standard deviation) or associated estimates of uncertainty (e.g. confidence intervals)
☐	☒	For null hypothesis testing, the test statistic (e.g. F, t, r) with confidence intervals, effect sizes, degrees of freedom and P value noted<br>*Give P values as exact values whenever suitable.*
☒	☐	For Bayesian analysis, information on the choice of priors and Markov chain Monte Carlo settings
☒	☐	For hierarchical and complex designs, identification of the appropriate level for tests and full reporting of outcomes
☒	☐	Estimates of effect sizes (e.g. Cohen's d, Pearson's r), indicating how they were calculated

Our web collection on statistics for biologists contains articles on many of the points above.

Software and code

Policy information about availability of computer code

Data collection	Two separate groups of rats, both with the same CA1 recording sites used in part for an internal replication, were recorded during the sequence memory task for three consecutive sessions with ~300 test trials per session. Throughout each experiment wide-band data was acquired automatically using digital headstages (32-channel, 25kHz sampling rate) and OmniplexD systems (256-channel, up sampled to 40kHz, Plexon, inc.) coupled to an automated behavioral rig run with custom MATLAB scripts which will be made available upon request. The OmniplexD systems also acquires 32 digital behavioral event inputs, 16 analog event inputs, and digital video (80fps) aligned to a single timing board. Voltage signals recorded from silicon probes and stainless steel wires were referenced to a stainless steel ground screw positioned over the cerebellum (low cutoff = 0.7 Hz). Local field potentials (LFP) and single-unit bands were separated into two data streams (LFP: 0.7 Hz–300Hz).
Data analysis	MATLAB (V2016a; V2021a) scripts using native functions were used with (e.g., 'mscohere', 'trapz','envelope'), the Chronux toolbox (http://chronux.org/), and circ_stats toolboxes for all analysis. Behaviorally-relevant events were identified through MATLAB and with simultaneous video tracking using DeepLabCut for all experiments.

For manuscripts utilizing custom algorithms or software that are central to the research but not yet described in published literature, software must be made available to editors and reviewers. We strongly encourage code deposition in a community repository (e.g. GitHub). See the Nature Portfolio guidelines for submitting code & software for further information.

Data

Policy information about availability of data

All manuscripts must include a data availability statement. This statement should provide the following information, where applicable:

- Accession codes, unique identifiers, or web links for publicly available datasets
- A description of any restrictions on data availability
- For clinical datasets or third party data, please ensure that the statement adheres to our policy

The datasets generated during and/or analyzed during the current study are available from the corresponding author on reasonable request. See data availability statement

Human research participants

Policy information about studies involving human research participants and Sex and Gender in Research.

Reporting on sex and gender	N/A
Population characteristics	N/A
Recruitment	N/A
Ethics oversight	N/A

Note that full information on the approval of the study protocol must also be provided in the manuscript.

Field-specific reporting

Please select the one below that is the best fit for your research. If you are not sure, read the appropriate sections before making your selection.

☒ Life sciences ☐ Behavioural & social sciences ☐ Ecological, evolutionary & environmental sciences

For a reference copy of the document with all sections, see nature.com/documents/nr-reporting-summary-flat.pdf

Life sciences study design

All studies must disclose on these points even when the disclosure is negative.

Sample size	For all experiments, a power analysis was performed to obtain sample sizes estimating moderate to large effects sizes (60-90% power, G*power calculator) using preliminary/pilot data.
Data exclusions	Rats with incorrect placement of the optic fiber or stainless-steel wires, improper injection site or viral expression, or defective electrodes (n = 1) were not included in the analysis.
Replication	All experiments were conducted with many internal replications. Two separate memory experiments were conducted (two separate groups of rats). Each experiment we included probes in the slm layer of CA1 and we directly compared these to find similar results. Additionally, we repeated the experiment three times in each rat and report similar results. We analyzed LFPs using multiple signal processing approaches (e.g., spectrograms, raw traces, filtered traces, envelope analysis, burst detection algorithms, etc.). While each analysis provided a useful perspective on the rhythmic activity in the brain, each approach replicated the main findings on the role of beta bursts in memory (and ruled out theta harmonic issues). In the optogenetic experiments, we repeated experiments on three different sessions and replicated the main results with varied stimulation frequencies and varied stimulation shapes. Each approach and stimulation pattern replicated the main effect that reuniens neurons (that project the slm layer of CA1) quite simply drive delta-beta synchrony in the prefrontal-hippocampal network.
Randomization	Upon arrival at the animal facility rats were randomly placed into each experiment and given IDs. Rats were then randomly chosen for implant/AAV surgeries using MATLAB (randperm function).
Blinding	Data collectors were not blinded to experimental groups because both experiments (open field/opto experiments and sequence memory task experiments) were fully automated using Plexon and MATLAB scripts and conducted in a space isolated from the experimenter and/or conducted as a within subjects design. This removed any need for user intervention throughout the session and greatly limits the possibility of experimenter bias. The behavioral states and conditions were automatically assessed using MATLAB and DeepLabCut tracking software (Mathis Lab, MA, USA) using two simultaneous video cameras. Additionally, the data were collected by multiple researchers. Data analysis was conducted blind via MATLAB scripts.

Reporting for specific materials, systems and methods

We require information from authors about some types of materials, experimental systems and methods used in many studies. Here, indicate whether each material, system or method listed is relevant to your study. If you are not sure if a list item applies to your research, read the appropriate section before selecting a response.

Materials & experimental systems

n/a	Involved in the study
☐	☒ Antibodies
☒	☐ Eukaryotic cell lines
☒	☐ Palaeontology and archaeology
☐	☒ Animals and other organisms
☒	☐ Clinical data
☒	☐ Dual use research of concern

Methods

n/a	Involved in the study
☒	☐ ChIP-seq
☒	☐ Flow cytometry
☒	☐ MRI-based neuroimaging

Antibodies

Antibodies used	Primary: Anti-GFP, Rockland catalog number 600-401-379, lot number 41686, 1:500 dilution<br>Secondary: Anti-Rabbit secondary, DyLight™ 488, VectorLabs, DK-8818, lot number ZE1017, 1:500 dilution
Validation	Per Rockland website: There are 924 publications citing this product, validating its use in rodents. This antibody has also been reported and validated by the Antibody Registry (https://antibodyregistry.org/search?q=600-401-379). The manufacturer provided a certificate of analysis for the Lot number used. Internal validations were also based on expected morphological expression patterns for reuniens neurons that project to the hippocampus which have been mapped in our lab and many others.<br>Per Vectorlabs website: There are over 55 publications citing this product. Company also provided Certificate of Analysis with a PASS specification validating the product.

Animals and other research organisms

Policy information about studies involving animals; ARRIVE guidelines recommended for reporting animal research, and Sex and Gender in Research

Laboratory animals	Long-Evans, ~1 year old
Wild animals	Study did not involve wild animals
Reporting on sex	Although male and female rats were used, sex was not evaluated as a variable since we have previously demonstrated there are no significant differences in performance between males and females on the sequence memory task, or by estrous cycle (Jayachandran et al., 2022).
Field-collected samples	Study did not involve field-collected samples
Ethics oversight	All experimental procedures using animals were conducted in accordance with the Florida International University Institutional Animal Care and Use Committee (FIU IACUC).

Note that full information on the approval of the study protocol must also be provided in the manuscript.
